# Insight in Adhesion Protein Sialylation and Microgravity Dependent Cell Adhesion—An Omics Network Approach

**DOI:** 10.3390/ijms21051749

**Published:** 2020-03-04

**Authors:** Thomas J. Bauer, Erich Gombocz, Markus Wehland, Johann Bauer, Manfred Infanger, Daniela Grimm

**Affiliations:** 1 Clinic for Plastic, Aesthetic and Hand Surgery, Otto-von-Guericke-University Magdeburg, D-39120 Magdeburg, Germany; thomas.bauer@med.ovgu.de (T.J.B.); markus.wehland@med.ovgu.de (M.W.); manfred.infanger@med.ovgu.de (M.I.); dgg@biomed.au.dk (D.G.); 2 Melissa Informatics, 2550 Ninth Street, Suite 114, Berkeley, CA 94710, USA; egombocz@ix.netcom.com; 3 Max Planck Institute of Biochemistry, D-82152 Martinsried, Germany; 4 Department of Biomedicine, Aarhus University Hospital, DK-8000 Aarhus C, Denmark; 5 Department of Microgravity and Translational Regenerative Medicine, Otto von Guericke University, Pfälzer Platz, 39106 Magdeburg, Germany

**Keywords:** linked open database, graphical SPARQL, semantic knowledgebase, proteome, integrin, cadherin

## Abstract

The adhesion behavior of human tissue cells changes in vitro, when gravity forces affecting these cells are modified. To understand the mechanisms underlying these changes, proteins involved in cell-cell or cell-extracellular matrix adhesion, their expression, accumulation, localization, and posttranslational modification (PTM) regarding changes during exposure to microgravity were investigated. As the sialylation of adhesion proteins is influencing cell adhesion on Earth in vitro and in vivo, we analyzed the sialylation of cell adhesion molecules detected by omics studies on cells, which change their adhesion behavior when exposed to microgravity. Using a knowledge graph created from experimental omics data and semantic searches across several reference databases, we studied the sialylation of adhesion proteins glycosylated at their extracellular domains with regards to its sensitivity to microgravity. This way, experimental omics data networked with the current knowledge about the binding of sialic acids to cell adhesion proteins, its regulation, and interactions in between those proteins provided insights into the mechanisms behind our experimental findings, suggesting that balancing the sialylation against the de-sialylation of the terminal ends of the adhesion proteins’ glycans influences their binding activity. This sheds light on the transition from two- to three-dimensional growth observed in microgravity, mirroring cell migration and cancer metastasis in vivo.

## 1. Introduction

Many types of human tissue cells split into two populations when cultured under simulated or real microgravity conditions. One population continues to grow in a monolayer (AD cells). The other one detaches from the bottom of a culture flask and forms three-dimensional aggregates called multicellular spheroids (MCS) [1]. This in vitro process appears to mirror metastasis in vivo, which also includes cell detachment and re-aggregation [2,3,4]. In recent studies, we subjected thyroid and breast cancer cells and endothelial cells, which had been cultured for a few days on ground, in Space or on a Random Positioning Machine (RPM) simulating microgravity, to mass spectrometry or microarray gene analyses. These high throughput omics technologies unveiled thousands of proteins or genes [5,6,7,8], including gravity-sensitive cell adhesion proteins such as integrins, which form alpha-beta dimers [9,10], cadherins [6,11,12], and other cell adhesion molecules [13,14,15] as well as CD44 [16], whose sensitivity to microgravity has repeatedly been described [17]. CD44 is a cell surface receptor that plays a role in cell-cell interactions, cell adhesion, and migration, helping the cells to sense and respond to changes in the tissue microenvironment [18,19].

The omics technology revealed changes of expression and/or accumulation of adhesion proteins under microgravity in AD and MCS cells as compared to control cells cultured under 1*g*-conditions [5,6]. Hence, additional information was collected about microgravity-dependent posttranslational modifications [20], including phosphorylation [9,21], ubiquitination [22,23], or isgylation [6,24], and about the intracellular translocation of adhesion proteins [25,26].

Posttranslational modification (PTM) is accomplished by enzymes such as kinases, phosphatases, or ligases [20,21,22,23,24]. In some types of PTM, carbohydrates are bound to reactive oxygens (O-glycan) or nitrogens (N-glycan) of a protein’s amino acid side chains [27]. In many cases, a monosaccharide coupled to an amino acid is extended towards the environment of a cell by a number of additional sugar monomers forming a chain, which may be branched or linear [28]. Carbohydrate units coupled directly to an amino acid can be determined by mass spectrometry [29]. The results of such analyses have been collected in overview databases such as the UniProt (https://www.uniprot.org/) or dbPTM databases (http://dbptm.mbc.edu.tw) [20]. However, very often this knowledge is not sufficient, because the biological effect of a protein-bound carbohydrate system is exerted by carbohydrate monomers located at the terminal end of a glycan system consisting of tens or even hundreds of carbohydrate monomers. This is true regarding the antigenicity of soluble or cell-bound proteins as well as regarding negative surface charges located at a cell surface and influencing the environment [28,30]. Both traits are often formed by sialic acids (SAs) that are located at the most distant end of a carbohydrate system bound to a protein [31]. Hence sialylation deserves further elucidation.

SA is a nine-carbon acidic monosaccharide [32], whose carboxyl group is dissociated at physiological pH [32,33] and generates a negative charge. SA is bound to the tip of glycan systems, if CMP-sialic acid is available [34]. This substance is built up along a well-known pathway with sialic acid synthase (NANS) as the key enzyme [35]. Linked to a CMP, SA is transported to its destination point, where it is transferred to the tip of an existing glycan structure [36]. In humans, the transfer is catalyzed by 20 different types of sialyltransferases [34,37]. Conversely, four types of human neuraminidases, also called sialidases, can catalyze removing SAs from existing carbohydrate systems [38,39].

SA molecules bound to proteins affect the half-life of many circulating glycoproteins and also influence the cell-cell communication, cell matrix interaction, and cell adhesion [40,41]. As microgravity has effects on cell adhesion [5,6] and also influences the sialylation activity of insect cells [42], we were interested to see whether microgravity influences the sialylation of human cells, affecting their adhesion behavior.

**Figure 1:**
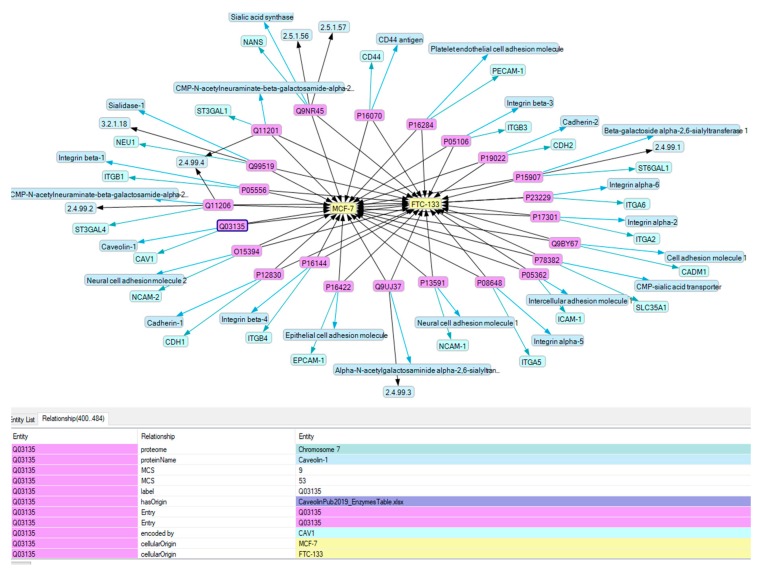
The initial semantic knowledge base used for the semantic enrichment of experimental data with current knowledge about the sialylation of adhesion proteins. The genes and names of human adhesion proteins found in omics experiments were loaded in a first step.

To address this question, we selected human adhesion proteins, which we found to play a role in experiments when cancer or endothelial cells changed their behavior during exposure to microgravity [5,6,25], and we used the Knowledge Explorer (KE) [43,44], which allows the enrichment of experimental data with knowledge from several fact and reference databases (Figure 1), if the names and related analysis results of the selected proteins were imported via their UniProt accession numbers into an initial resource description framework (RDF) to create a knowledge base (KB) through a combination of semantic protocol and RDF query language (SPARQL) searches across several databases and importing their results iteratively (Figure 1). The resulting knowledge base was then assessed in detail to evaluate the importance of our findings in context. This data revealed that the sialylation of adhesion proteins influences the adhesion cells’ behavior. 

## 2. Results and Discussion

### 2.1. Selected Cell Adhesion Proteins and Their Characterization Regarding Sialylation

**Figure 2:**
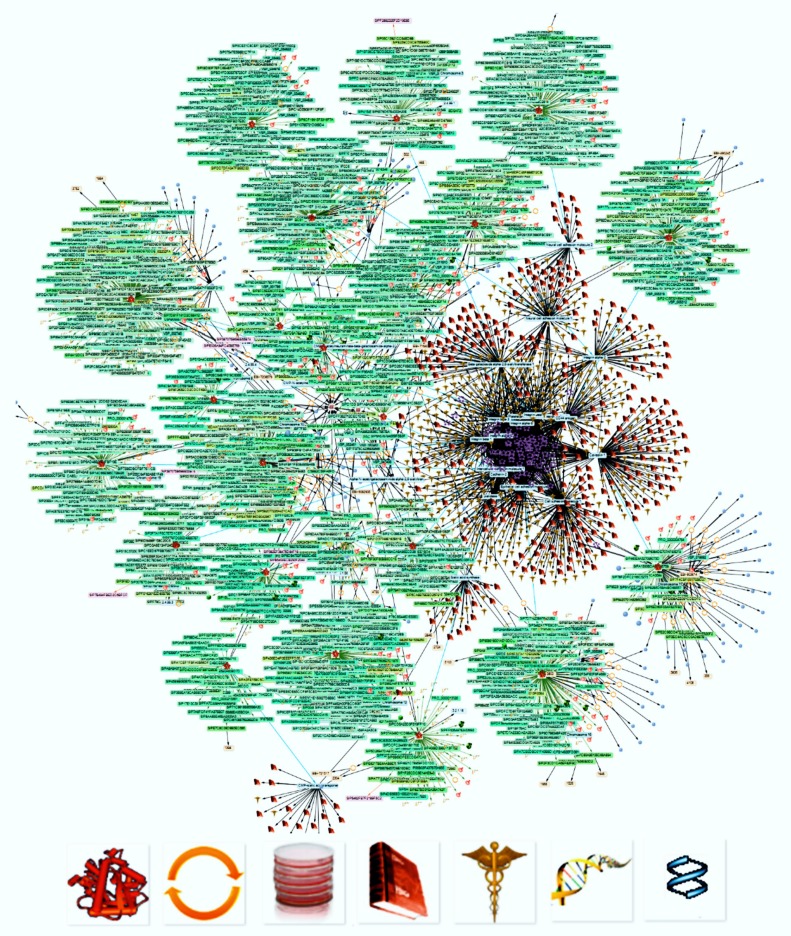
The results of KE-mediated searches for scientific information in reference and fact databases. The SwissProt accession numbers of the proteins shown in Table 1 were used to search for manuscripts about their sialylation in combination with further traits regarding their structures, genes, sequences, and functions. The various icons shown in the bottom line at a high magnification indicate the type of nodes (from left: protein structures, functions, cells producing the proteins, publications, diseases wherein the proteins play a role, genes, and sequences). The text within the picture becomes legible after magnification.

Aiming to get information about the function of SAs bound to the tips of the glycans, which cell adhesion proteins bear, we selected the proteins shown in Table 1. Information about them obtained by the earlier omics analysis and detected in the UniProt database was incorporated in a comprehensive KB. Using this KB to selectively retrieve references revealed more than 200 publications about the sialylation of these proteins. In addition, information about the identified proteins was collected regarding their structures, genes, sequences, and functions, as well as the types of cells producing them and the diseases where they play a role (Figure 2). Evaluating the references revealed 17 membrane-inserted proteins bearing glycans with SAs at their tips (Table 1).

#### 2.1.1. Cadherins and Other Cell-Cell Adhesion Proteins

CDH1 proteins were detected in MCF7 cells and CDH2 proteins in FTC-133 cells (Table 1). According to studies published in the literature, they occur in a sialylated and de-sialylated form: Human MCF7 breast cancer cells and pancreatic ductal adenocarcinoma cells (PDAC) have e-cadherins bearing glycans with terminal SAs [46,47,48]. Changes in the terminal SA units influence the e-cadherin’s adhesion capability [46,48]. In addition, N-cadherins expressed by various tumor cells bear glycans with terminal SAs [49,50,51]. Melanoma cells show sialylated N-cadherins, which have an influence on the metastatic capabilities of these tumor cells [50]. Removing the sialic acids from N-cadherin of HeLa cells by sialidase affects N-cadherin-ZO-1 association [49]. Furthermore, VE-cadherins of endothelial cells are sialylated. SAs of endothelial cells of human umbilical cord veins are suggested to play a role in VE-cadherin organization [52]. Their degree of sialylation is regulated by TNF-alpha [53]. We found VE-cadherin protein in EA.hy926 cells [7]. In these cells, the expression of *CDH5* mRNA is reduced when they form tubular structures under microgravity [54]

CADM1 (cell adhesion molecule 1) is another membrane protein mediating homophilic cell-cell adhesion. We found CADM1 in MCF7 cells and with a higher concentration in FTC-133 cells (Table 1). Moreover, CADM1 is sialylated in A549 lung cancer cells [55]. Our proteome analysis also unveiled the junctional adhesion protein A (JAM-A) in MCF7 and FTC-133 cells. In both cell lines, a significant influence of microgravity was not detectable (Table 1). However, these proteins may bear N-glycans with terminal sialic acids, which regulate the cells’ (CHO cells) adherence [56].

#### 2.1.2. Integrins

In the MCF7 cell line, exposure to microgravity significantly decreased ITGB4 in MCS cells, while it increased ITGA5 in these cells as compared to 1*g* control cells and AD cells, respectively (Table 1). Furthermore, ITGB1 was enhanced in AD cells as compared to 1*g* control cells and MCS cells. In the literature, a considerable amount of information was found indicating that integrins bear SAs, which affect the adhesion capabilities of cells [57].

A β1 integrin region, called the β1 I-like domain, is important for ligand binding. This region carries N-glycans at three asparagine residues (Asn 192, Asn 249, and Asn 343). Their terminal galactose may or may not be elongated by α2-6 sialic acid [58]. In the desialylated form, binding to the ligand is stronger than in the sialylated one [59]. The effect of sialylation appears to be due to conformational changes of the integrin β1 protein [58]. The conformational changes may be responsible for the following observations made on the in vivo behavior of various cells: HD3 colonocytes regulate their invasion and migration via the sialylation of their β1-integrins [60]. Sialylated integrin β1 of SW480 colon cancer cells supports cell binding to fibronectin and counteracts apoptosis by activating paxillin and AKT [61]. Human SW48 colon epithelial cells show 2–6 sialylation of the β1 integrin. When enhanced quantities of α2-6 sialic acids were bound to the SW480 cells’ β1-integrin subunits, their aggressiveness was especially high [62]. In this case, the SA blocks the pro-apoptotic effects of secreted galectin 3 [63]. The sialyltransferase inhibitor Lith-*O*-Asp decreased the sialic acid modification of integrin β1 in mice and inhibited the expression of phospho-focal adhesion kinase, phospho-paxillin, and the matrix metalloproteases MMP2 and MMP9 [64]. Another sialyltransferase inhibitor, AL10, reduces the sialylation of various integrin molecules, especially β1 integrin, and attenuates the activation of the integrin downstream signaling mediator focal adhesion kinase [65]. 

The alteration of the sialylation of integrin-β1 also influences its dimerization with α-integrin subunits [58]. MDA-MB-231 cells bear sialylated integrin α2/β1. In these cells, changes of the degree of sialylation affect adhesion without alteration of integrin expression [66]. Moreover, C4-2B prostate cancer cells exhibit α2β1 integrins on their cell surface with sialylated α2 subunits. The linked sialic acid residues are required for the cell adhesion to collagen type I and are responsible for the interaction with the carbohydrate moiety of an asialylated ganglioside, which changes the integrin’s activity [67]. Furthermore, pancreatic Capan-1 cells bear sialylated α2β1 integrins on their surface. The content of SAs of the glycans of this protein influences cell adhesion and invasion of these cancer cells by enhancing FAK-tyr-397 phosphorylation [48].

Another integrin dimer detected on cancer cells is α5β1- integrin. For this dimer, the structure of the integrin alpha linked sialylated glycan has already been identified [68]. α5β1-integrins are also expressed by melanoma cells [69]. In these cells, both integrin subunits are sialylated and influence the binding behavior of the dimer. Simultaneously, they influence the invasion capability of the cells, but do not affect the fibronectin binding of the isolated proteins. Hepatocellular carcinoma cells have α5β1-integrins, which bind to fibronectin. If fibronectin binding is not possible, they bind to galectin 1, which induces apoptosis. If the α5β1-integrins are α2,6-sialylated, interaction with Gal-1 and anoikis is prevented [70]. If β1 integrin of the α5β1-integrin dimers is sialylated, it regulates β1 integrin subunit binding to fibronectin and induces cellular activities [71], while sialylation of the α5 subunit is important for the proper formation of the heterodimer [72]. After removal of sialic acids, α5β1-integrins of human melanoma G361 cells failed to bind to fibronectin-conjugated Sapharose [73]. Moreover, human T24 bladder cancer cells showed α3β1-integrins [74]. α6β1-integrin was found on endothelial cells mediating the cells’ interaction with CD151 of platelets. The interaction promotes angiogenesis and depends on sialic acids because it is abolished by neuraminidase [75]. 

In addition to ITGB1, an influence of sialylation on ITGB4 and ITGB3 activities is described in the literature. The sialylation of cell surface integrin β4 was down-regulated during the epithelial-mesenchymal transition of human keratinocyte HaCaT cells [31]. In colon cancer cells, NEU1 decreased the sialylation of integrin β4 and suppressed cell adhesion in vitro [66,76]. When human WM793 primary melanoma cells gain metastatic competence, they show increased levels of sialylated αVβ3 integrins [77]. Cooperation of PECAM-VEGFR2-integrin β3 is significantly reduced when integrin β3 lacks 1,6-sialic acid [78].

#### 2.1.3. Cell Adhesion Molecules

In the literature and fact databases, a number of studies were found indicating that the sialylation of cell adhesion molecules plays a decisive role, when endothelial cells, brain cells, or cancer cells break and reestablish external contacts. For example, the stabilization of ICAM-1 via sialylation decreases metastatic ability on colorectal cancer cells [79]. 

In our proteome studies, ICAM-1 was detected in FTC-133 cells at a very low concentration, but not in MCF7 cells. In addition, we have found NCAM-1 in thyroid cancer cells and NCAM-2 in MCF7 cells. In neither case did we find a significant influence of microgravity on the accumulation of these proteins (Table 1). However, neural cellular adhesion molecules (NCAMs) show a peculiar sialylation pattern. In contrast to many other sialylated adhesion proteins, which have one or two terminal SAs at the tip of their glycans [80], NCams often have terminal carbohydrate polymers consisting of up to 100 SA monomers [81,82]. Polysialic acid is characteristic for developing neurons, but it is also expressed by glucagonomas [83]. Such terminal SA polymers modify NCAM and regulate cell adherence and cell-cell interaction [84,85]. In neuroblastoma cells, as well as in small cell lung cancer, it favors metastasis [86,87]. In chick embryonic brain cells, the partial and total desialylation of NCAMs regulates cell adhesion differently [88]. In MCF7 cells, polysialylation affects the adhesion of NCAM to the extracellular matrix [89]. Furthermore, polysialylated NCAM reduces E-cadherin-mediated cellular adhesion, facilitating the dissemination of tumor cells [90]. EpCAM (epithelial cell adhesion molecule) is another homophilic interaction molecule of epithelial cells. We detected this molecule in MCF7 cells. Chen et al. found that EpCAM is sialylated in MCF7 and that its degree of sialylation may be changed by drugs [91]. 

We found PeCAM in the membrane fractions of Ea.hy926 and microvascular endothelial cells [7]. Its electrophoretic behavior suggested a lower pI, as indicated in databases. The difference may be explained as PeCAM occurs in sialylated and de-sialylated forms [92]. In endothelial cells, SA regulates the adhesion and downstream apoptotic signaling [93]. In human mesothelioma cells, a PECAM 1 was found that is bound to Asn-25 and bears a terminal α2,3 sialic acid moiety. This negatively charged moiety forms an electrostatic bridge with the positively charged Lys-89, stabilizing the PECAM-1 homophilic binding interface [94]. Depending on the status of sialylation, PECAM-1 influences the process of angiogenesis in different ways [92,95].

#### 2.1.4. CD44 Antigen

The CD44 antigen bears terminal SAs at the tips of its glycans [96]. If CD44 N-glycans bear terminal SAs, hyaluronan binding is blocked as competing intramolecular contacts of sialic acid with arginine side chains are formed [97]. The sialidase inhibitor 2-deoxy-2,3-dehydro-N-acetylneuraminic acid blocks the recognition of hyaluronic acid, showing that sialylation negatively regulates CD44 activity [98]. Activation of sialidase by TNFalpha via MAPK38 initiates binding of CD44 to hyaluronic acid [97]. In colorectal cancer, metastasis to the liver occurs more frequently when CD44 is expressed on the surface together with sialyl Lewis(a) antigens [99]. Matsubara et al. concluded from their study that the sialylation status of CD44 may be more important for binding to hyaluronic acid than its degree of expression [100]. 

#### 2.1.5. Interaction of Sialylated Proteins

**Figure 3:**
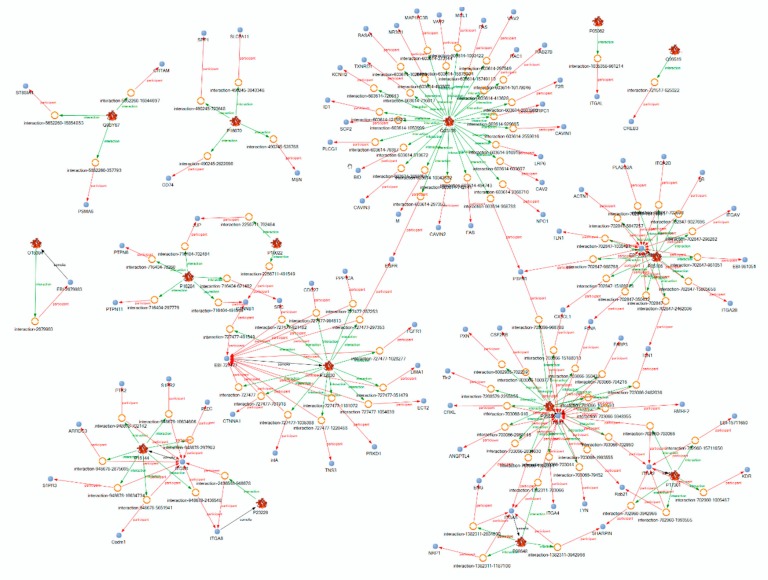
The interaction partners of the proteins shown in Table 1. The participants in the interaction were restricted to proteins playing a role in the signaling of cell adhesion. The text within the picture becomes legible after magnification.

The 17 proteins shown in Table 1, as well as two endothelial cell proteins, were found sialylated in the studies, underlying the references found by KE searches. In order to show how the sialylation of these proteins could trigger the cell reactions described in the studies mentioned above, we performed an interaction study using the KB. Figure 3 shows the interaction partners of the membrane-inserted proteins selected. They include a number of proteins, SPP1, MSN, FAS, CAV2, RAC1, SRC, EGFR, TLN1, ACTN1, PXN, PLEC, PTK2 and KDR, which emerged in our earlier studies as important players in transducing the signals of cell adhesion to the cytoskeleton [5,6,45,54,101,102]. 

### 2.2. Enzymes Responsible for the Status of Adhesion Proteins

Some proteins shown in Table 1 are significantly changed in quantities (e.g., CDH1, EPCAM, CD44) but others (e.g., JAMA or various integrin subunits) not when exposed to microgravity. This suggests that besides the accumulation of sialylable proteins, their actual sialylation could play a role [58,103]. Taking the information described in Section 2.1 together, it appears evident that sialylation and de-sialylation occurs on equal proteins. From this observation, one may conclude that the amount of sialic acid transiently bound to a protein is regulated by an interplay of sialyltransferases and neuraminidases [104,105,106]. Our proteome analysis revealed NEU1 (Table 2). Its accumulation is more than 20-fold higher in FTC-133 cells than in MCF7 cells. In both cases, however, its concentration was higher in MCS cells than in AD cells found after incubation under simulated microgravity (Table 2). 

We also detected NANS in MCF7 breast cancer and FTC-133 follicular thyroid cancer cells during recent proteome analyses. In MCF7 cells, much more NANS was found as compared to the FTC-133 cells. Exposure of the cells to microgravity slightly enhanced the NANS concentrations in both cell lines (Table 2). 

Although the CMP-sialic acid transporter coded by the *SLC35A1* gene emerged in the proteome analysis of MCF7 cells (Table 2), neither the literature nor a semantic analysis of the functional association of the proteins of Table 1 indicated a role of this transporter in the sialylation of adhesion proteins. In addition, three types of sialyltransfereases were detected (Table 2). However, ST3GAL1 (CMP-N-acetylneuraminate-beta-galactosamide-alpha-2,3-sialyltransferase 1; Q11201), ST3GAL4 (CMP-N-acetylneuraminate-beta-galactosamide-alpha-2,3-sialyltransferase 4; Q11206), and ST6GALNAc2 (Alpha-N-acetylgalactosaminide alpha-2,6-sialyltransferase 2; QUJ37) were found in MCF7 cells, while only ST3GAL4 emerged in the analysis of the FTC-133 cells (Table 2). These enzymes are mainly involved in the sialylation of mucins [107]. Still, recent publications described that ST3GAL1 contributes to the sialylation of integrin β1 and CD44 [108] and that the silencing of ST3GAL4 impairs the Ccl5-triggered integrin activation of mouse myeloid cells [109]. 

**Figure 4:**
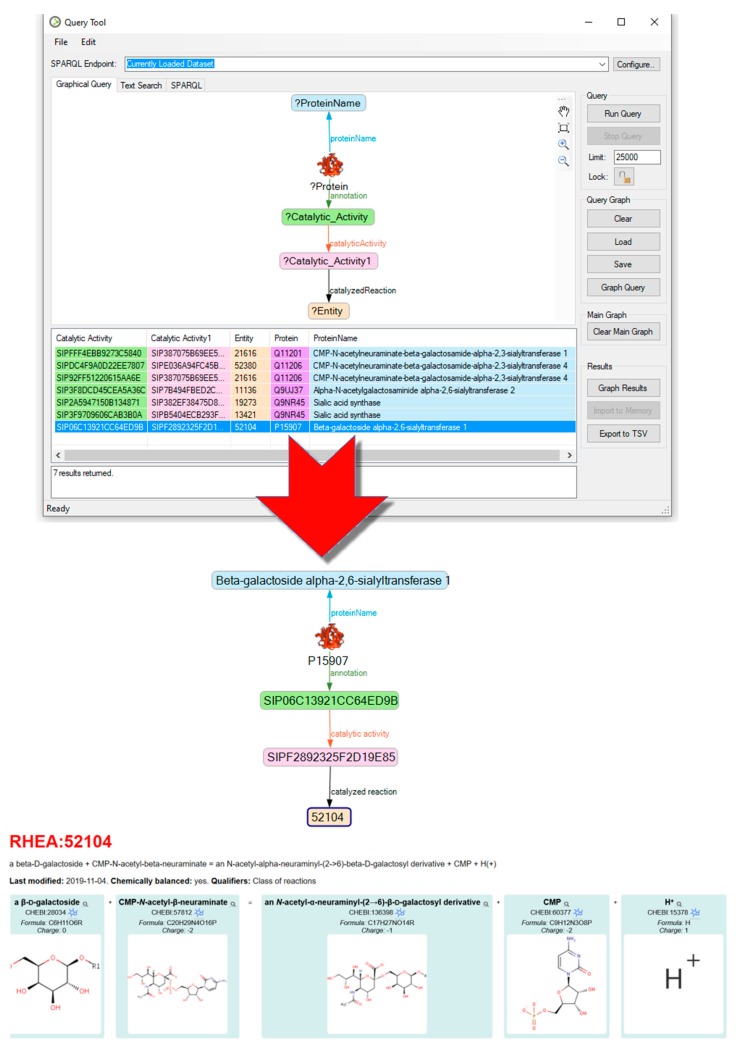
Graphical query for searching enzyme-catalyzed reactions catalyzed in Rhea (https://www.rhea-db.org/reaction?id=?). The in dark blue highlighted result for P15907 in the query can be brought onto canvas, where the link from Rhea 52104 depicts the reaction catalyzed by ST6GAL1. SA is transferred from SA-CMP to a terminal galactose of an existing glycan (see: https://www.rhea-db.org/reaction?id=15907).

Although sialyltransferase ST3GAL1 and ST3GAL4 were found in our proteome experiments, ST6GAL1 (Beta-galactoside alpha-2,6-sialyltransferase 1) is most often mentioned within the manuscripts retrieved for this study about sialylation of adhesion proteins [110]. As shown in Figure 4, it transfers sialic acid from CMP-sialic acid to galactose-containing acceptor substrates. ST6GAL1 is capable of binding sialic acids to the tips of 391 different glycan structures, (see: https://www.glygen.org/glycoprotein_search.html) which have a galactose at their terminal end. 

**Figure 5:**
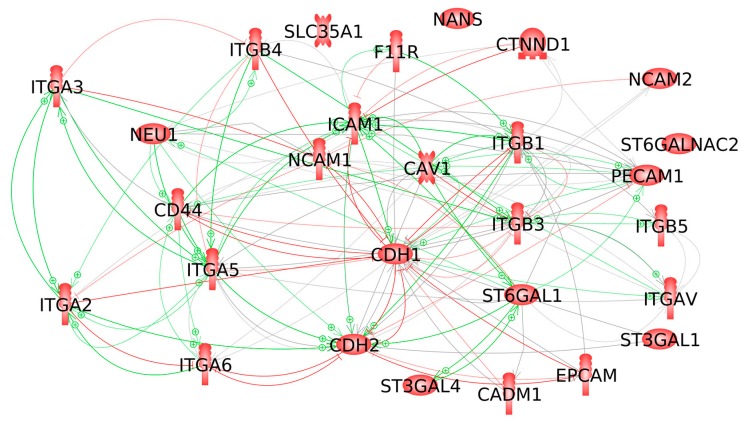
The mutual influence of selected entities at gene expression. Genes whose proteins were selected for a detail analysis by the network approach are shown. The green arrows indicate activating and the red arrows inhibiting effects. The grey lines indicate that interactions take place within the entities whose effects have not yet been clarified. The interaction network was built up using Elsevier Pathway Studio plus.

Proteins sialylated by ST6GAL1 are integrin beta 1 [110,111,112,113,114,115] and integrin beta3 [78]. ST6Gal I links SA to integrin β1 in α2-6 mode [116]. Furthermore, in lung cancer cells, A549 CADM1 is sialylated by ST6Gal1. The sialylation is triggered by miRNA-199a and initiates signals to ErbB2/Erbb3 [55]. ICAM-1 is stabilized via sialylation by ST6Gal1 on colorectal cancer cells. The stabilization decreases metastatic ability [79]. PeCAM, which we found in an earlier mass spectrometry analysis of Ea.hy926 and microvascular endothelial cells [7], undergoes changes in sialylation as it is sialylated by ST6Gal 1 and desialylated by NEU1 [92]. Hence, the literature suggests that ST6GAL1, according to the current knowledge, plays a very important role in the sialylation of adhesion proteins. This suggestion is supported by interaction analyses, as shown in Figure 5.

#### 2.2.1. Regulation of Quantities

As shown above, the status of the sialylation of adhesion proteins depends on the interplay of active sialyltransferases and neuraminidases. In vivo, this interplay can be affected by growth factors, drugs and other physiological components [53,64,65,76,95,115,116]. The expression of neuraminidases is affected by current physiological and pathological conditions [117,118] and has, e.g., an influence on cell apoptosis [119]. Sialyltransferase activity can be changed by growth factors [120,121], oncogenes [60,122], or microRNAs [123] within a given type of cells. 

In addition, the entities selected for this study have a considerable mutual influence on a gene expression level. Figure 5 shows that ST6GAL1 and NEU 1 not only sialylate or desialylate target glycans, but also influence the expression of the proteins bearing the SAs. The most interesting mRNAs of ST6GAL1 and of ST3GAL4 are up-regulated together [124], and the regulation of *ST6GAL1* and *CDH2* is interconnected [125]. Furthermore, sialidase treatment of human lung epithelial cells influences the expression of integrin α5 [126]. In FTC-133 and MCF-7 cells, the concentration of ST6GAL1 protein was obviously below the detection threshold of the applied mass spectroscopy technique. However, a microgravity-dependent regulation of its gene became evident when FTC-133 thyroid cells returned from the Shenzhou 8 Space flight were investigated [8]. In this experiment, the microarray gene analysis revealed a great number of genes which were more than two-fold up- or down-regulated in FTC-133 cells returning from the Shenzhou-8 spaceflight. The *ST6Gal 1* gene was amongst them. Its expression was 9.38-fold down-regulated in AD cells and 8.12-fold in MCS cells. Therefore, one may conclude that microgravity can influence the sialylation of adhesion proteins via the regulation of the mRNA expression of ST6GAL1. 

#### 2.2.2. Regulation of Activities

In mouse hepatocarcinoma cells, caveolin-1 (CAV1) favors the sialylation of the α5 subunit promoting α5β1 integrin-dependent cell adhesion via enhancing FAK-mediated signaling [127]. Nonetheless, CAV1 can also support the activities of sialidase [128]. Thus, the expression and accumulation of caveolin-1 in AD cells (cells remaining adherent during exposure to microgravity) is most interesting [129,130,131], as AD cells of the FTC-133 cell line show enhanced CAV-1 [130], while AD cells of the MCF7 cell line show a reduced CAV1 (Table 2). Moreover, catenin delta 1 can also play a role. It is abundantly present in MCF7 and FTC-133 cells. However, less of this protein was detected in MCS cells formed under microgravity as compared to AD and 1*g* control cells (Table 2). When endothelial cells achieve confluence, catenin delta 1 binds sialidase to PECAM-1 (CD31) to desialylate its glycan [95]. This suggests that not only do the quantities of neuraminidases and sialyltransferases decide whether sialylation or desialylation is favored, but so do their location and the kind of membrane association, respectively.

### 2.3. Biochemical and Biophysical Effects of SAs Present on Extracellular Domains of Surface Proteins

Many effects on cell behavior have been observed when the sialylation of adhesion proteins changed in vivo and in vitro. But little is known about the links between the sugar moieties bound to the tips of extracellular glycan of adhesion molecules and the effects exerted by these negatively charged molecules. Some clues can be derived from the literature, pointing to the possibilities that the conformation of the proteins, to which the sialic acid is bound, is changed [58]. A change of the conformation has an influence on the binding to extracellular matrix proteins or to extracellular domains of surface proteins of other cells. It could also give different signals to the cell interior with direction towards the cell nucleus [45]. 

It remains of interest whether the electrostatic units generated by the negative charges of the sialic acid have an effect under in vivo conditions when cells are embedded in a tissue and are surrounded by extracellular matrix. Until now, electrostatic units firmly bound to cell surfaces could be observed and quantitatively determined when singularized tissue cells were placed in an electric field of direct current [132,133]. It is known that the electrophoretic behavior of the cells depends on the amount of sialic acids bound to the cell surface [134], and similarities between the behavior of cells in an electric field and on an RPM have recently been reviewed [135]. In both cases, adhesion proteins played a major role. 

## 3. Materials and Methods

### 3.1. Biological Experiments

#### 3.1.1. Cancer Cell Proteins

The proteins were obtained by mass spectrometry from FTC-133 human follicular thyroid carcinoma cells and from MCF7 human breast adenocarcinoma cells according to protocols described in [5,6]. Prior to analysis, both types of cells had been grown either within a monolayer under normal 1*g* laboratory conditions or exposed to an RPM, where one part remained adherent (AD cells), while the other one formed three-dimensional aggregates (MCS cells). Monolayer cells cultured under 1*g*, AD cells, and MCS cells were harvested and pelleted in separate samples. Each sample was subjected to a proteome analysis. In total, 12 different cell samples, i.e., four per incubation condition, were analyzed to determine as many proteins as possible of the MCF7 cells; and five pellets were investigated to identify proteins of the FTC-133 cells.

Mass spectrometry was performed, as described earlier in detail [5,6]. Shortly, cells were lysed. Their proteins were digested overnight at 37 °C with endoproteinase Lys-C (Wako Chemicals GmbH, Neuss, Germany). The digested peptides were purified and then separated using the Thermo easy n-LC 1000 system (Thermo Scientific, Waltham, MA, USA). The peptides eluting from the column were directly sprayed into a Q Exactive HF mass spectrometer (Thermo Scientific, Waltham, MA, USA) via a nano-electrospray ionization source (Thermo Scientific, Waltham, MA, USA) [136,137]. The mass spectrometer was operated in a data-dependent top 15 mode. Survey scans and fragmentation scans were acquired at resolutions of 60,000 and 15,000 respectively (*m*/*z* = 200). Fragmentation was performed on precursors isolated within a window of 1.4 m /z with a normalized collision energy setting of 27. 

Raw data from the mass spectrometer were processed using MaxQuant computational proteomics platform version 1.5.2.22 (Computational Systems Biochemistry, Max-Planck-Gesellschaft, Munich, Germany) [138] using the standard parameters. At least 5900 different proteins were identified in five FTC-133 cell samples [5], and 6500 different proteins were found in 12 MCF-7 cell samples [6]. These proteins formed the base for applying the MaxLfQ algorithm to determine the relative protein concentration by delayed normalization, as explained by Cox et al. in detail [139]. This label-free quantification technology is based on the assumption that a majority of proteins exists which does not change between the samples of a cell line. 

#### 3.1.2. Endothelial Cell Proteins

The proteins were obtained by mass spectrometry from EA-hy926 and dermal microvascular endothelial cells according to protocols described in [7]. Shortly, both types of cells had been grown either within a monolayer under normal 1*g* laboratory conditions or exposed to an RPM, where one part remained adherent (AD cells) while the other one formed three-dimensional tubular aggregates (tube cells). Monolayer cells cultured under 1*g*, AD cells, and tube cells were harvested and pelleted in separate samples. Each cell sample was lysed by sonication. Then, soluble proteins and remaining cell fragments were separated by centrifugation. Both fractions were subjected to free flow electrophoretic separation. The resulting fractions were subjected to SDS page and stained. Bands of interest were cut out and forwarded to mass spectrometry, which was performed using an UltiMate 3000 nano-LC system (Dionex, Idstein, Germany) coupled to an ESI-linear ion trap (LTQ XL, Thermo Electron, Karlsruhe, Germany), as described in [140]. 

#### 3.1.3. Cancer Cell Genes

The differential gene expression on FTC-133 thyroid cancer cells was analyzed, after the cells had been cultured in space for 10 days during the Shenzhou-8 space mission. For the gene arrays, 1*g* control cells and both AD cells and MCSs returning from the 10-day lasting spaceflight were collected separately for RNA extraction. Each time, four independent RNA preparations from the resulting three different conditions were processed and hybridized with the Illumina HumanWG-6_V2_0_R3_11223189_A array (Illumina, Inc., San Diego, CA, USA), as described in [8].

### 3.2. Creation of A Semantic Network 

To create a semantic network, harmonize the content from multiple resources, and allow for graphical querying and reasoning, experimental data were imported to establish an initial resource description framework (RDF) knowledge base using KE (Melissa Informatics, Berkeley, CA, USA—former IO Informatics). This tool allows one to create, merge, and/or align semantic knowledge bases (SKB) in the form of RDF serializations as files or in backend databases and to configure and connect them to public semantic protocol and RDF query language (SPARQL) endpoints for query and import [20,43,44,45]. Its import functions provide abilities for nomenclature alignment, to set or automate reification IDs for blank nodes, and to establish mapping for spreadsheets or XML datasets. After importing the experimental proteomics data, an initial partial ontology imported from UniProt (in XML-RDF format) was transformed into a core RDF representation. A thesaurus manager was used to harmonize synonyms and avoid duplication during the import process.

**Figure 6:**
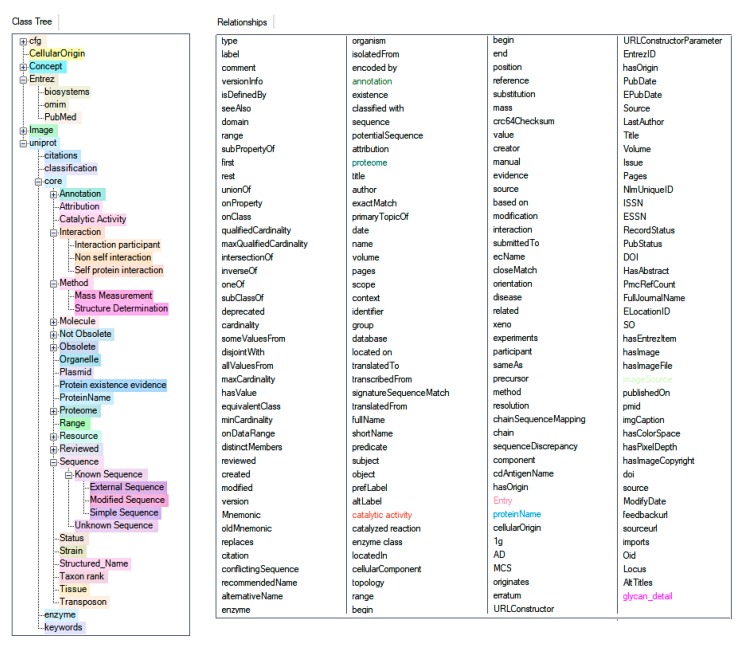
The dynamic generated applications ontology and relationships in the KE Knowledge Graph. Left panel: class tree with partially expanded subclasses; right panel: relationships between class entities. The colorization of the classes allows for easy distinction of their instances in the knowledge graph. All Entrez databases are displayed in light brown shades. Subclass colors are shades of the color of the corresponding root class.

UniProt content was queried for each of the proteins using its SPARQL endpoint [43,44,141]. This added functional, cellular location, chromosomal, and interaction properties to augment the information on the enzymes and reported protein functions [45,142]. The information collected was used to retrieve content from the Entréz resources OMIM, Protein, PubMed, and Biosystems (Figure 6), as well as from dbPTM, GlyGen, Rhea, and Atlas of Genetics and Cytogenetics [20,143] by means of graphical queries and their result imports (Figure 4).

### 3.3. Interaction Analysis

To investigate and visualize the interactions between the selected proteins and genes, we inputted relevant UniProtKB entry numbers into the Pathway Studio plus software (Elsevier Research Solutions, Amsterdam, the Netherlands). The identified genes were analyzed according to their mutual regulation [54]. 

### 3.4. Statistical Evaluation

The Man-Whitney-U-Test was used to compare the LfQ values of MCF7 cells harvested after incubation either at 1*g* or at a microgravity condition. Data is presented as the mean ± standard deviation (SD) with a significance level of *p* < 0.05.

## 4. Conclusions

The omics network approach described in this paper shows how to gain advantage from combining the results of three different experiments with each other and with the knowledge described in the literature, while focusing on those parts of the data which are related to the topic of cell adhesion. The data experimentally obtained on the accumulation of adhesion proteins in three different types of cells by mass spectrometry [5,6,7] and by microarray studies on mRNA expression [8] were incorporated in a common network with the current knowledge on sialylation. This semantic network generation was made possible with the help of KE, an advanced information retrieval tool for generating and interrogating complex knowledge graphs. Taking all this information together, we were able to conclude that microgravity influences the sialylation-dependent activities of adhesion proteins on three levels: (i) expression and accumulation of adhesion proteins, (ii) expression and accumulation of enzymes sialylating or de-sialylating glycan systems bound to adhesion proteins, and (iii) expression, accumulation, and localization of scaffold proteins supporting the activity of the enzymes. The gained knowledge suggests that the process of linking cells to each other or to the ECM under microgravity includes the sialylation of extracellular domains of adhesion proteins. 

Hence, it appears worthwhile to emphasize the sialylation of adhesion proteins in future space research, as the related transition from a two- to a three-dimensional growth appears to mirror some steps of cell migration and cancer cell metastasis in vivo where ST6GAL1 plays a role [144].

## Figures and Tables

**Table 1:** Proteins of MCF7 and FTC-133 cancer cells cultured under normal gravity (1*g*) or under simulated microgravity as adherent (AD) or as aggregated cells (MCS). The proteins were selected from recent proteome analyses [5,6] because their sialylation is described in the literature. UniProt accession numbers, genes, and label free quantitation values (LfQ) are indicated.

Protein	Gene	MCF-7			FTC-133		
		1*g*	AD	MCS	1*g*	AD	MCS
P19022	*CDH2*	0	0	0	16.8	19.1	20.4
P12830	* CDH1	40.8 ± 3.5	33.5 ± 4.4	19 ± 4.5	0	0	0
P05362	*ICAM-1*	0	0	0	1.9	0	1.26
P13591	*NCAM-1*	0	0	0	54.7	50.5	44.7
O15394	*NCAM-2*	2 ± 0.5	1.7 ± 0.5	2.1 ± 0.5	0	0	0
P16422	*EPCAM*	4.85 ± 1.9	10.27 ± 2.9	3.61 ± 1.9	0	0	0
Q9BY67	* CADM1	0.85 ± 0.3	0.5 ± 0. 4	0	4.9	4.5	6.15
Q9Y624	*JAMA*	2.55 ± 0.6	2 ± 1.4	3.4 ± 3.9	3.04	0	2.54
P16070	* CD44	12.3 ± 1.3	3.8 ± 0.8	8.4 ± 2.2	31.8	71.4	63.6
P05556	* ITGB1	15.1 ± 1.7	22.8 ± 3.8	16 ± 2.7	208	173	136
P05106	*ITGB3*	0	0	0	5.31	3.44	4.3
P16144	* ITGB4	7.4 ± 0.7	6.1 ± 1.4	3.1 ± 0.6	0	0	0
P06756	* ITGAV	7.4 ± 0.6	10.1 ± 2.5	8.6 ± 2.7	108	106	87.5
P17301	* ITGA2	4.5 ± 0.3	5.8 ± 1.4	4.2 ± 0.4	3.36	4.04	3.24
P26006	* ITGA3	1.4 ± 0.5	1.3 ± 0.9	0.8 ± 0.3	113	75.2	59.2
P08648	* ITGA5	0.95 ± 0.2	1.5 ± 0.4	2.88 ± 0.7	24.9	36.2	28.3
P23229	* ITGA6	0.8 ± 0.05	0.47 ± 0.05	0.49 ± 0.13	8.03	10	9.9

LfQ (label free quantitation) are scores given as × * 10^8^ [20]; Each LfQ value shown for the MCF-7 cells is an average value of the independent measurements of four equally prepared samples. Entities labeled by * have already been mentioned in refs. [6] or [45]. They are included for comparison and completion purposes, because they are sialylated.

**Table 2:** Proteins of MCF7 and FTC-133 cancer cells cultured under normal gravity (1*g*) or under simulated microgravity as adherent (AD) or as aggregated cells (MCS). The proteins were selected from recent proteome analyses [5,6] because they are involved in the sialylation of proteins. Uniprot accession numbers, genes, and label free quantitation values (LfQ) are indicated.

Protein	Gene	MCF-7			FTC-133		
		1*g*	AD	MCS	1*g*	AD	MCS
Q9NR45	*NANS*	69.5 ± 4.7	107 ± 29	93.2 ± 14	7.34	8.9	11.5
P78382	*SLC35A1*	0.29 ± 0.2	0	0.34 ± 0.26	0	0	0
Q11201	*ST3GAL1*	0.2 ± 0.06	1.05 ± 0.5	0.71 ± 0.31	0	0	0
Q11206	*ST3GAL4*	0	0.15 ± 0.18	0.33 ± 0.39	0.53	0	0
Q9UJ37	*ST6GALNac2*	0.18 ± 0.08	0	0.12 ± 0.03	0	0	0
Q99519	*NEU1*	0.97 ± 0.3	0.24 ± 0.36	0.78 ± 0.15	23.5	28.7	38.2
O60716	* CTNND1	39.5 ± 1.5	39.4 ± 8.9	19.3 ± 4.5	125	103	82.6
Q03135	* CAV1	13.2 ± 1.7	3.34 ± 0.43	8.66 ± 1.6	64.7	92.5	53.5
P15907	*ST6GAL1*	0	0	0	0	0	0

LfQ (label free quantitation) are scores given as × * 10^8^ [20]; Each LfQ value shown for the MCF-7 cells is an average value of the independent measurements of four equally prepared samples [6]. Entities labeled by * have already been mentioned in ref. [6] or [45]. They are included for comparison and completion purposes because they influence the sialylation of adhesion proteins.

## Abbreviations

ST3GAL1	CMP-N-acetylneuraminate-beta-galactosamide-alpha-2,3-sialyltransferase 1
ST3GAL4	CMP-N-acetylneuraminate-beta-galactosamide-alpha-2,3-sialyltransferase 4
ST6GALNac2	Alpha-N-acetylgalactosaminide alpha-2,6-sialyltransferase 2
ST6GAL1	Beta-galactoside alpha-2,6-sialyltransferase 1
SLC35A1	CMP-sialic acid transporter
NEU1	Sialidase-1
SA	Sialic acid
NANS	Sialic acid synthase
CAV1	Caveolin-1
CDH2	Cadherin-2
CDH1	Cadherin-1
CADM1	Cell adhesion molecule 1
ICAM-1	Intercellular adhesion molecule 1
NCAM-1	Neural cell adhesion molecule 1
NCAM-2	Neural cell adhesion molecule 2
PECAM-1	Platelet endothelial cell adhesion molecule 1
EPCAM	Epithelial cell adhesion molecule
JAMA	Junctional adhesion molecule A
CTNND1	Catenin delta 1
CD44	CD44 antigen
ITGB1	Integrin beta-1
ITGB3	Integrin beta-3
ITGB4	Integrin beta-4
ITGA2	Integrin alpha-2
ITGA5	Integrin alpha-5
ITGA6	Integrin alpha-6
RDF	Resource description framework (RDF)
SPARQL	Semantic protocol and RDF query language (SPARQL)
SKB	Semantic knowledge base
LfQ	Label free quantification
RPM	Random positioning machine
AD cells	Cells growing adherently on the RPM
MCS cells	Cells growing within multicellular spheroids on the RPM
